# Functionality of potato virus Y coat protein in cell-to-cell movement dynamics is defined by its N-terminal region

**DOI:** 10.1128/jvi.01400-25

**Published:** 2025-11-20

**Authors:** Anže Vozelj, Tjaša Mahkovec Povalej, Katja Stare, Magda Tušek Žnidarič, Katarina Bačnik, Valentina Levak, Ion Gutiérrez-Aguirre, Marjetka Podobnik, Kristina Gruden, Anna Coll, Tjaša Lukan

**Affiliations:** 1 Department of Biotechnology and Systems Biology, National Institute of Biology 54766, Ljubljana, Slovenia; 2 Jožef Stefan International Postgraduate School, Ljubljana, Slovenia; 3 Department of Molecular Biology and Nanobiotechnology, National Institute of Chemistry 68913, Ljubljana, Slovenia; Tsinghua University, Beijing, China

**Keywords:** potato virus Y, potato (*Solanum tuberosum*), coat protein (CP), viral movement, point mutations

## Abstract

Potato virus Y (PVY) is one of the top 10 economically most important plant viruses and responsible for major yield losses. We previously suggested the involvement of the N-terminal region of PVY coat protein (CP) in PVY spread. By constructing different N-terminal deletion mutants of the PVY N605 strain, we here show that deletions of 40 or more amino acid residues from the N-terminal region of the CP resulted in the PVY multiplication limited to primary infected cells in *Nicotiana clevelandii* plants. Deletion of 26 residues profoundly impaired PVY cell-to-cell movement and prevented systemic PVY spread, while deletions of 19-23 residues allowed delayed systemic PVY spread. Introduced point mutations in the identified region prevent (S21G) or delay (G20P) PVY movement. In summary, this work shows the significance of the CP N-terminus for movement of the PVY.

**IMPORTANCE:**

Potato virus Y (PVY) is one of the most economically important plant viruses worldwide, since it causes major yield losses in Solanaceae, especially in potato, where it is the causal agent of potato tuber necrosis ringspot disease, negatively impacting tuber quality and significantly reducing potato yield. By constructing different PVY N-terminal deletion mutants, we identified the regions of the coat protein that are important for efficient PVY cell-to-cell movement. Understanding these regions, responsible for cell-to-cell as well as long-distance movement, is crucial for economically important plant viruses such as PVY, since dissolving such complex processes will enhance our understanding of the PVY infective cycle and contribute to the development of effective tackling strategies against PVY infections in potato and prevent yield losses.

## INTRODUCTION

Plants undergo constant exposure to various pathogens and pests, with viruses being the most severe amongst them. Potato virus Y (PVY) is classified among the top 10 economically most important plant viruses infecting solanaceous crops including potato, tomato, tobacco, and pepper (1). PVY is the causal agent of potato tuber necrosis ringspot disease (2), a devastating disease that negatively impacts potato tuber quality and yield (3).

PVY belongs to the *Potyvirus* genus. Its genome consists of 9.7 kb positive sense single-stranded RNA (+ ssRNA) encoding for single open reading frame (ORF) that is translated into 350 kDa long polyprotein which is cleaved into 10 mature proteins (3). Furthermore, PVY genome contains an additional ORF *pipo* (“pretty interesting *Potyviridae* ORF”), embedded within the P3 cistron of the polyprotein. Expression of *pipo* occurs through transcriptional slippage by the viral RNA polymerase and results in production of the P3N-PIPO fusion protein, which is involved in cell-to-cell movement (4, 5).

The majority of potyviral proteins are multifunctional and they altogether contribute to the establishment of successful viral infection that encompasses multiple steps including transmission by aphids, penetration of viral particles into the cell, virion disassembly, translation, replication, suppression of host defense mechanisms, virion assembly, and virus movement from the primary infected cells to neighboring cells and systemic spread (6).

Viral movement is facilitated through plasmodesmata, plant-specific structures connecting two adjacent cells that serve as gateways for cell-to-cell movement and contribute to the establishment of systemic infection (7–10). At least four potyviral proteins, coat protein (CP), cylindrical inclusion protein (CI), P3N-PIPO, and the second 6 kDa protein (6K2) protein are essential for cell-to-cell movement of potyviruses (11). P3N-PIPO is localized in the plasmodesmata and directs CI to form conical structures that are crucial for assistance of potyviral intercellular movement through plasmodesmata, which was shown for turnip mosaic virus (TuMV) in *Nicotiana benthamiana* (12). Accumulation of CP in plasmodesmata during infection is also crucial since together with the potyviral Helper-Component Proteinase (HCPro) it increases the size exclusion limit (SEL) of plasmodesmata, which was determined by microinjection studies for bean common mosaic necrosis potyvirus in *N. benthamiana* (13). Systemic infection encompasses viral movement through phloem and less often through xylem to reach and infect plant distant tissues (14, 15). However, before reaching vasculature, plant viruses need to cross various cellular barriers including bundle sheath, vascular parenchyma, and companion cells, to reach sieve elements, through which they are transported to distant tissues (14). The invasion of distant tissues requires unloading from sieve elements into companion cells and cell-to-cell movement into bundle sheath and mesophyll cells (14). Despite PVY being one of the most extensively studied potyviruses on a molecular scale, information about crucial factors governing cell-to-cell as well as systemic spread is scarce (6, 11).

The cryo-EM structures of some potyviruses including PVY, watermelon mosaic virus, and TuMV virions have been determined and offered a detailed insight into the conserved helical arrangement of CPs assembled around viral ssRNA as well as the conserved three-dimensional structure of CP among them (16–18). CP is the only potyviral structural protein and more than 2,000 copies of CP encapsidate the viral ssRNA molecule to form potyviral virions (6, 10). Individual CP is built of three distinct regions. The N-terminal region with residues up to Val44 in PVY, which are exposed at the outer surface of the virus, is flexible and thus its 3D structure was not determined. The rest of the N-terminal region, from Val44 to Gln76, is structured. It has an extended structure with one short alpha helix in the middle (17). The central core has a globular shape. The C-terminal region is in the lumen of the virus, and its extended structure is supported by the viral ssRNA scaffold (17). Beyond its primary role in viral encapsidation, the CP is indispensable for aphid transmission, potyviral RNA amplification, and cell-to-cell movement (10, 13, 19). Functional studies of the CP protein indicate crucial roles of its N- and C-terminal regions and the core region in potyvirus infectivity. In the case of PVY, the absence of the flexible CP N-terminal region hinders the formation of filaments and cell-to-cell viral movement (17). For soybean mosaic virus, the CP C-terminal region is involved in CP intersubunit interactions, viral cell-to-cell and long-distance movement, and virion assembly (20). In potato virus A (PVA), phosphorylation of the Thr243 residue on CP C-terminal region is crucial for PVA replication *in planta* (21). These results and results of several other studies suggest that different CP regions are important for cell-to-cell movement of potyviruses (22–27). It is not known yet in what structural shape the viruses are propagated through the plant, as assembled virions or viral ribonucleoprotein complexes associated with CP (14, 15).

The so far generated evidence-based knowledge differs from one potyvirus to another, and thus, more research is needed to comprehensively elucidate these complex processes, particularly for those economically relevant potyviruses such as PVY. By constructing different PVY N-terminal deletion mutants, we identified the regions of the CP that are important for efficient PVY cell-to-cell movement.

## RESULTS

### Deletion of 40 or more amino acid residues from the PVY CP N-terminal region affects cell-to-cell movement but does not prevent replication

**Fig 1:**
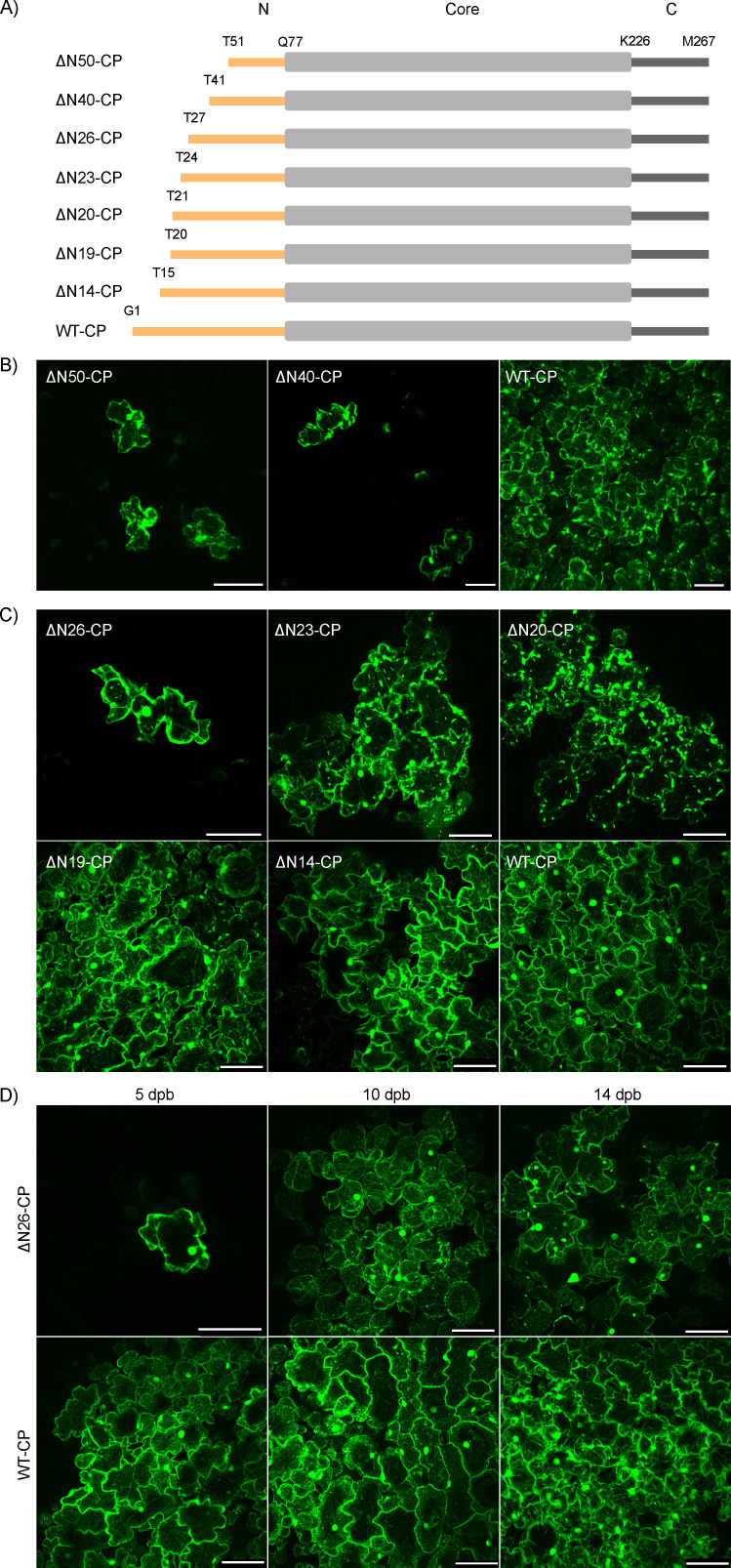
Amino acid deletions at PVY CP N-terminal region influence viral cell-to-cell movement. (**A**) Schematic representation of constructed mutants with deletions on PVY CP N-terminal region (N-terminal region, orange; core region, gray; C-terminal region, dark gray) with labeled amino acids. (**B**) Confocal microscopy images showing viral replication is limited to single cells when 50 (ΔN50-CP) or 40 (ΔN40-CP) amino acid residues were deleted at CP N-terminal region, while non-mutated clone (WT-CP) shows cell-to-cell spread 10–12 dpb. Scale bar: 100 µm. (**C**) Five dpb viral replication is limited to single cells in ΔN26-CP mutant, while cell-to-cell virus spread was observed for CP deletion mutants ΔN23-CP, ΔN20-CP, ΔN19-CP, and ΔN14-CP. Scale bar: 100 µm. (**D**) ΔN26-CP cell-to-cell spread was observed at later time points (10 and 14 dpb). Scale bar: 100 µm.

Previously, we showed that PVY with deleted 50 N-terminal amino acid residues was still able to replicate, but based on the low and spatially limited levels of RNA accumulation, we suggested that N-terminal region is required for cell-to-cell movement (17). To confirm this hypothesis, we here constructed two green fluorescent protein (GFP) tagged PVY (PVY N605-GFP) mutants lacking either 40 or 50 amino acid residues at the CP N-terminal region (hereafter ΔN50-CP and ΔN40-CP). In all constructed mutants, GFP was inserted between NIb and CP coding sequence, flanked by protease recognition sites, allowing GFP excision from the polyprotein after translation (28). We followed the spread of ΔN50-CP and ΔN40-CP with confocal microscopy upon bombardment of *Nicotiana clevelandii* leaves (Fig. 1B). Neither mutant was able to move cell-to-cell, remaining limited to bombarded cells (Fig. 1B). We detected viral RNA replication of both mutants in the bombarded leaves, albeit at significantly lower levels in comparison to the non-mutated clone (hereafter WT-CP) (Fig. S1 and Datasets S1 and S2). Despite the lower RNA levels observed in leaves bombarded with constructed mutants, confocal microscopy image analysis showed that the accumulation of GFP in individual infected cells was the same in WT and ΔN40-CP mutant, confirming unhindered replication (Table S2). These findings corroborate our previous results showing that N-terminal region CP truncations still allow viral replication (17) and further show that the absence of either 50 or 40 N-terminal residues abolishes cell-to-cell viral movement.

### The deletions of 19–26 amino acid residues of the PVY CP N-terminal region are influencing dynamics of viral cell-to-cell spread

To further assess which region of CP N-terminal is crucial for cell-to-cell virus spread, we constructed GFP-tagged mutants lacking 26, 23, 20, 19, and 14 amino acid residues at the CP N-terminal region (Fig. 1A) and followed their localization under confocal microscope at different time points after virus inoculation (Fig. 1C and D, Table S1, and Dataset S2). Mutants lacking 26 (ΔN26-CP) and 23 (ΔN23-CP) residues on CP N-terminal region had the first non-deleted amino acid replaced with glycine (G) during the mutagenesis.

At 5 days post-bombardment (dpb), ΔN14-CP and ΔN19-CP mutants did not show any differences in cell-to-cell spread compared to WT-CP, while a lower spread level was noticed for ΔN20-CP and ΔN23-CP (Fig. 1C). The most pronounced difference in comparison to other mutants and to WT-CP was observed in the case of ΔN26-CP mutant, which remained confined to single cells at five dpb (Fig. 1C and D). However, at later time points (10 and 14 dpb), in approximately half of the infected plants, we also observed cell-to-cell viral movement, although substantially delayed compared to WT-CP (Fig. 1D and Table S1). Also, the measured viral RNA load for ΔN26-CP mutant was significantly lower than that of WT-CP at all 5, 10, and 14 dpb (Fig. S2 and Dataset S1).

**Fig 2:**
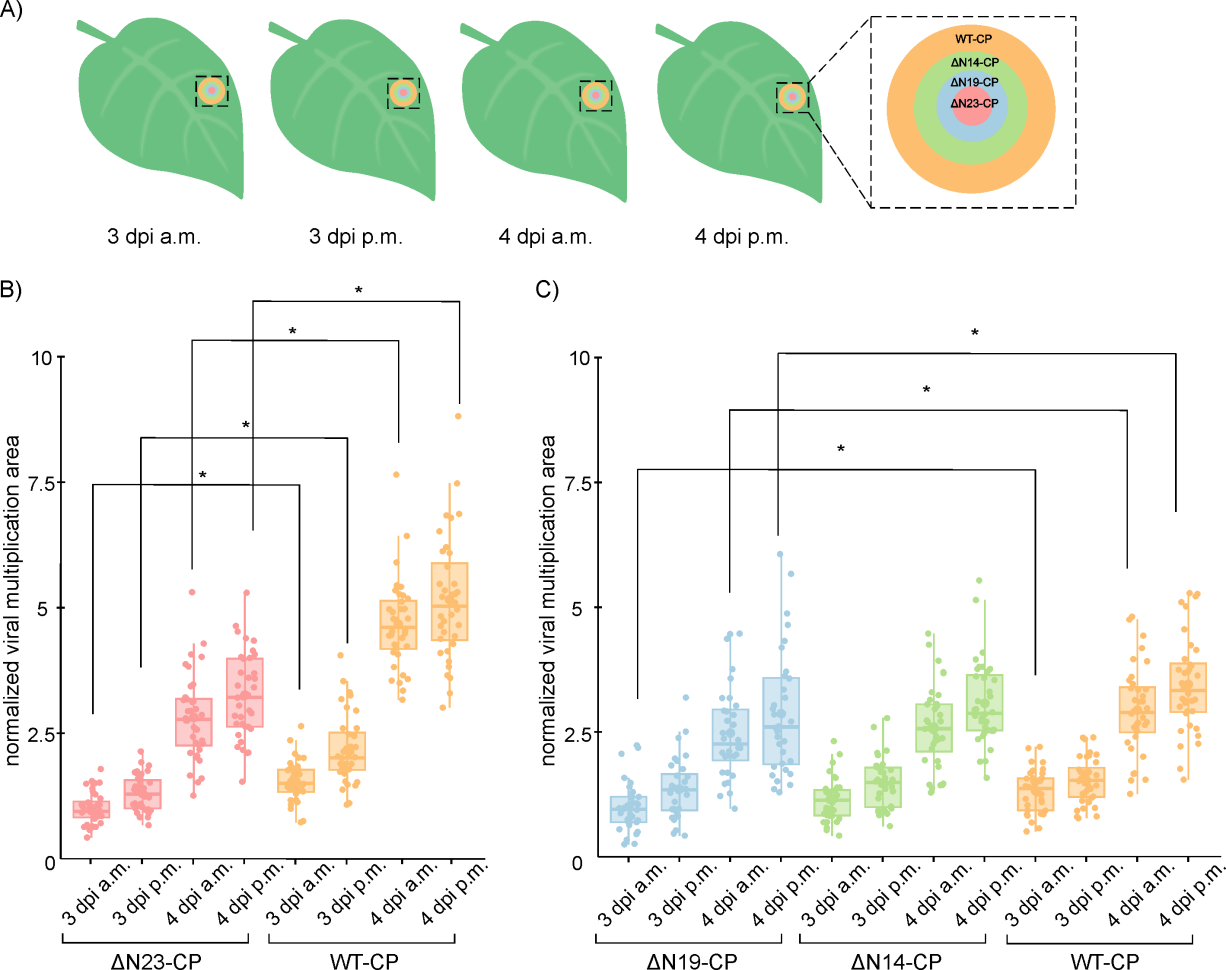
Precise analysis of the viral multiplication areas revealed a hampered cell-to-cell virus spread in ΔN19-CP and ΔN23-CP mutants. (**A**) The scheme of the viral multiplication area at different time points for all mutants compared with WT-CP. The area was followed by more precise analysis using whole plant imaging system for examining cell-to-cell virus spread of ΔN23-CP, ΔN19-CP, ΔN14-CP, and WT-CP in four time points including 3 dpi in the morning (a.m), 3 dpi in the afternoon (p.m), 4 dpi a.m. and 4 dpi p.m. Note that the viral multiplication area was the largest after infection with WT-CP and decreased in the latter order ΔN14-CP, ΔN19-CP, and ΔN23-CP. (**B and C**) Normalized viral multiplication area. After inoculating leaves with WT-CP or mutants, plants were imaged with whole plant imaging system. Images of three selected viral multiplication areas per leaf per plant were taken at four time points. Differences were statistically evaluated using Welch’s *t*-test. Results are presented as boxplots with dots representing normalized viral multiplication areas (Datasets S3 and S4) for ΔN23-CP, WT-CP (**B**) and ΔN19-CP, ΔN14-CP, WT-CP (**C**). Statistically significant differences (*P* < 0.05) are denoted with an asterisk (*). Vertical lines present all points except outliers. Raw and normalized data, number of plants, and statistics are specified in Datasets S3 and S4. The same trend was observed in a replicate experiment (Fig. S3 and Dataset S5). For comparison between two independent experiments, data measured for mutant viruses were normalized to median of values obtained for WT virus (Dataset S6).

Whole plant imaging was conducted to more precisely examine the dynamics of cell-to-cell virus spread of WT-CP, ΔN23-CP, ΔN19-CP, and ΔN14-CP through time on inoculated *N. clevelandii* leaves (Fig. 2A). As expected, the results revealed statistically significant differences in the viral multiplication area between N23-CP and WT-CP, and ΔN19-CP compared to WT-CP (Fig. 2B and C and Datasets S3 and S4). A similar, though less evident, trend was observed in the case of ΔN14-CP compared to WT-CP, which was not statistically significant (Fig. 2C and Dataset S4).

These results show that the dynamics of viral cell-to-cell spread is dependent on the length of the CP N-terminal region (Fig. 1 and Dataset S2). More precisely, as the number of CP N-terminal region deleted amino acid residues increases, cell-to-cell movement speed is decreased (Fig. 2B and C and Datasets S3, S4, and S6). We conclude that amino acids in the regions 19–26 on CP N-terminal region are essential for an optimal PVY cell-to-cell movement. Furthermore, with electron microscopy, we observed that viral assembly at all tested deletion mutants is feasible (Fig. S10).

### Systemic viral spread dynamics is affected by the cell-to-cell movement

**Fig 3:**
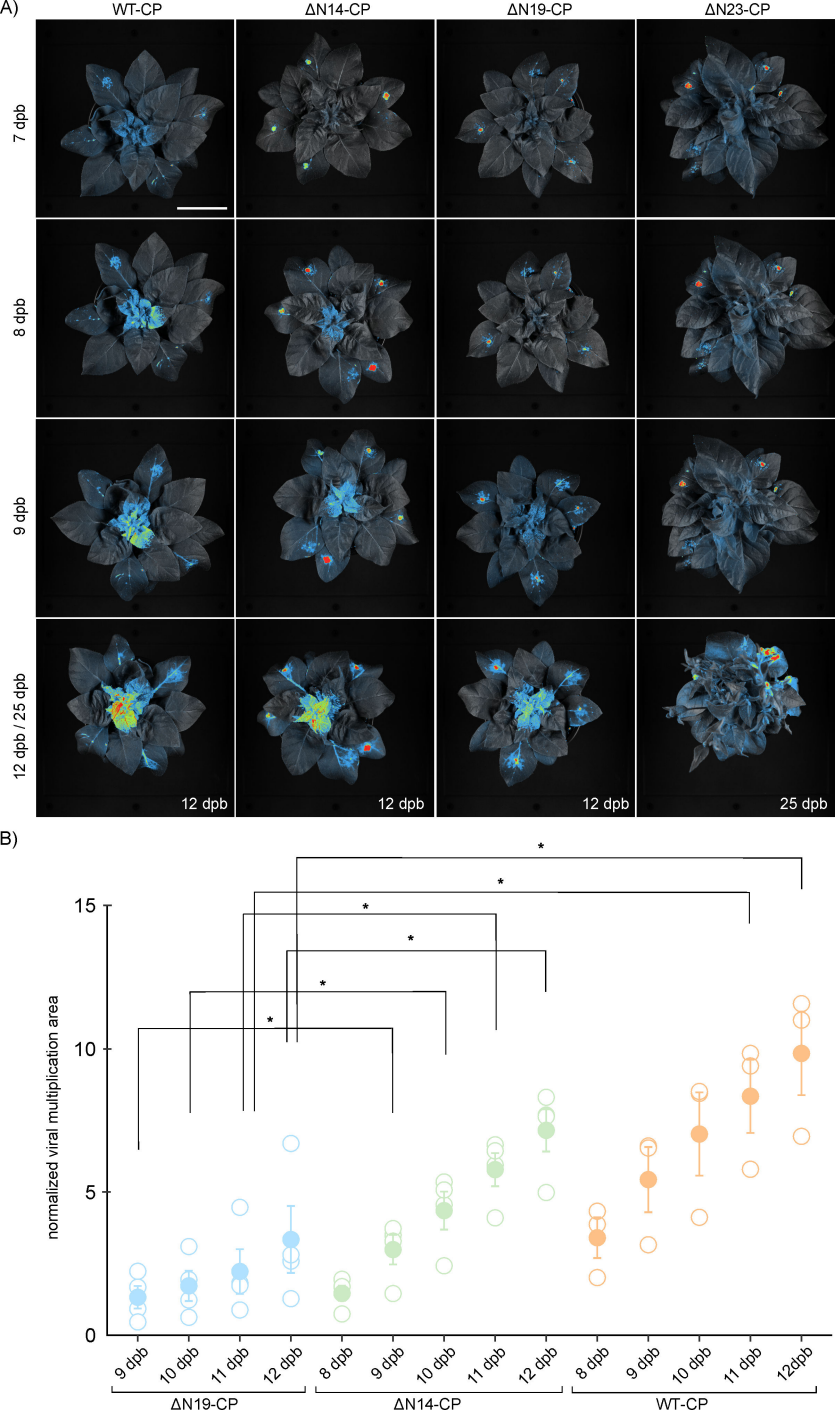
Systemic viral spread of mutants is delayed similarly to cell-to-cell movement. (**A**) Spatiotemporal PVY distribution in *N. clevelandii* systemic tissue. Viral multiplication area was followed 7–12 and 25 dpb, by using whole plant imaging system. Scale bar: 5 cm. (**B**) Quantification of viral multiplication area in the region of interest (ROI) in the *N. clevelandii* systemic tissue in total count per mutant in five selected time points including 8–12 dpb. Since ΔN19-CP did not spread systemically at 8 dpi, normalized viral multiplication area is not shown. Mean (represented with filled dot) and standard error are shown. Individual measurements are shown as empty dots, representing normalized viral multiplication area. Differences were statistically evaluated using Welch’s t-test. Statistically significant differences (*P* < 0.05) are marked with an asterisk (*). Raw and normalized data, number of plants, and statistics are specified in Dataset S7. Since ΔN26-CP is not capable of spreading systemically, it was not included in the analysis. Also, ΔN23-CP had such a delayed spread that it was not possible to do a quantification of viral multiplication area at 8–12 dpb (Fig. 3A).

Since viral cell-to-cell spread is a prerequisite for systemic viral spread, we further tested if the observed delay in cell-to-cell viral spread of mutants (Fig. 1C, 2B and C) affects the systemic viral spread. In contrast to the systemic spread of WT-CP, which is detectable already at seven dpb, the spread of ΔN23-CP, ΔN19-CP, and ΔN14-CP mutants to systemic leaves occurs with a delay (Fig. 3A). If compared to WT-CP, ΔN14-CP was observed in systemic tissue with 1 day delay (8 dpb), ΔN19-CP with 2 days delay (9 dpb), and ΔN23-CP with approximately 3 weeks delay (25 dpb) (Fig. 3A). The mutant ΔN26-CP did not reach systemic leaves even at the highest tested dpb (Fig. S4).

Quantitative analysis of viral multiplication signal in systemic tissue confirmed that WT-CP achieved the largest multiplication area, followed by ΔN14-CP and lastly ΔN19-CP in all observed time points (Fig. 3 and Dataset S7). When comparing ΔN14-CP and WT-CP, we observed a trend of slower spread in the case of ΔN14-CP, albeit not statistically significant (Fig. 3B and Dataset S7). ΔN23-CP was not included in the comparison, due to the considerable delay in the systemic spread exhibited by this mutant (Fig. 3A), which made it difficult to measure the viral multiplication area. In conclusion, systemic viral spread is abolished for ΔN26-CP and decreasingly hampered in the case of ΔN23-CP and ΔN19-CP, while the ΔN14-CP mutant is able to move similarly as WT-CP, albeit with a short delay.

### Substitution of serine with glycine on position 21 of CP N-terminal region abolished PVY cell-to-cell viral movement and virion assembly

**Fig 4:**
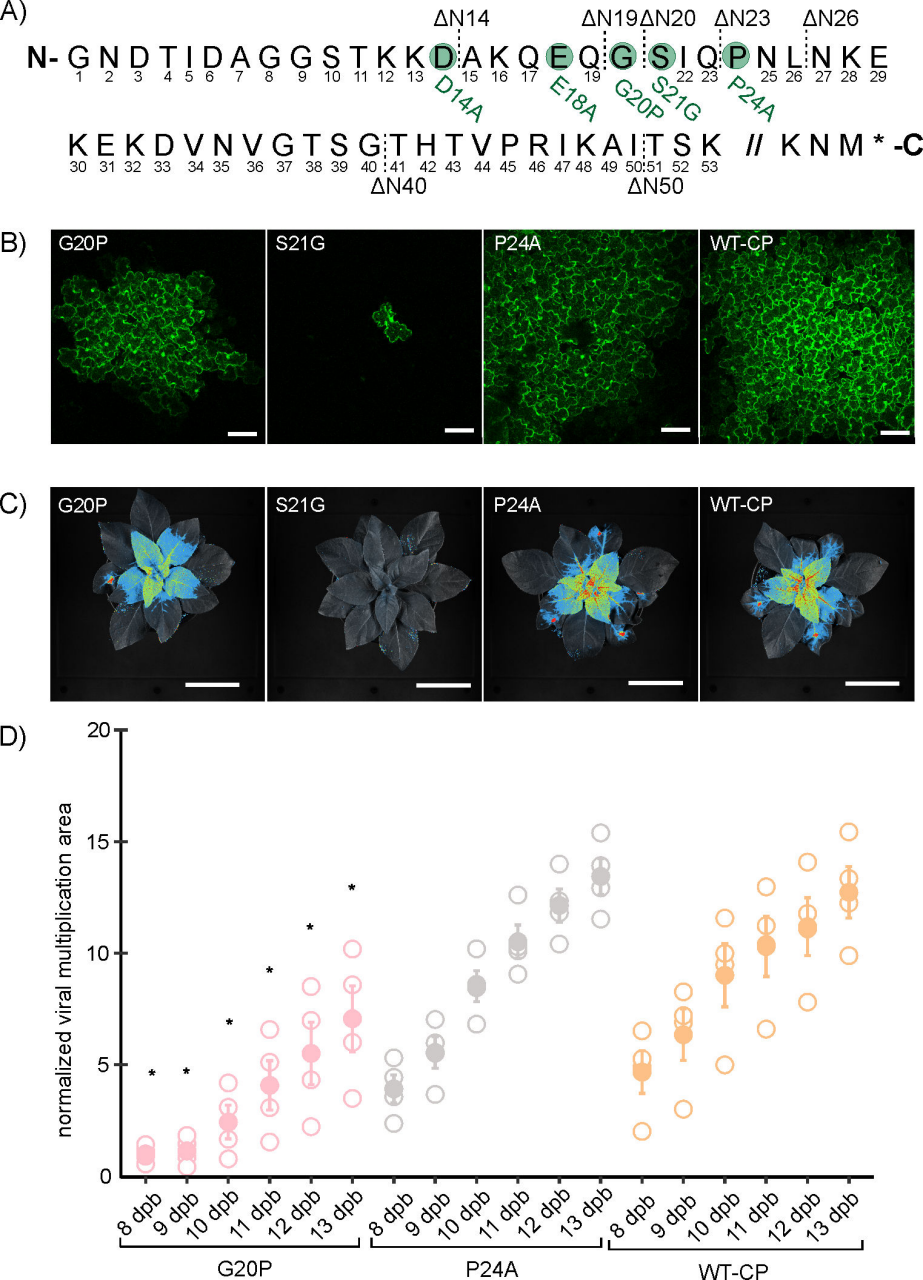
Substitution of serine to glycine on position 21 of CP N-terminal region abolished cell-to-cell viral movement. (**A**) Amino acid sequence of GFP-tagged infectious clone PVY-N605(123) CP protein with labeled constructed deletion and point mutants. The scheme presents mutants with deletions of 14 (ΔN14-CP), 19 (ΔN19-CP), 20 (ΔN20-CP), 23 (ΔN23-CP), 26 (ΔN26-CP), 40 (ΔN40-CP), or 50 (ΔN50-CP) amino acids on CP N-terminal region and mutants with point mutations including substitutions of negatively charged aspartic acid to alanine on position 14 (D14A), negatively charged glutamic acid to alanine on position 18 (E18A), glycine to proline on position 20 (G20P), serine to glycine on position 21 (S21G), and proline to alanine on position 24 (P24A). (**B**) Confocal microscopy images showing viral cell-to-cell spread of mutants G20P, S21G, P24A, and WT-CP 5 dpb. Scale is 100 µm. (**C**) Spatiotemporal PVY distribution for constructed point mutants G20P, S21G, P24A, and WT-CP in *N. clevelandii* systemic tissue as observed by whole plant imaging system 12 dpb. Scale is 5 cm. (**D**) Quantification of viral multiplication area in the upper systemic leaves of bombarded *N. clevelandii* in six selected time points (8–13 dpb). Mean (represented with filled dot) and standard error are shown. Individual measurements are shown as empty dots, representing normalized viral multiplication area. Differences between analyzed point mutants were evaluated with Welch’s *t*-test. Statistically significant differences (*P* < 0.05) between G20P mutant and WT-CP are represented with asterisks (*), while there were no statistically observed differences between P24A and WT-CP (see Dataset S8 for all results of statistical testing). Vertical lines present all points except outliers. The results were confirmed in additional experiments (Fig. S5 and Dataset S9).

To pinpoint amino acids important for cell-to-cell virus movement, we generated point mutations in the part of CP N-terminal region that was observed as being important for viral movement (Fig. 1 to 3). We point mutated charged residues Asp14, Glu18, potential phosphorylation site at Ser21, and 3D structure breakers Gly20 and Pro24. All, except Pro24, are highly conserved across sequenced PVY strains. At position 24, 50% of PVY strains carry Pro and the others Ser (Fig. S9). Selected residues were changed to Ala (D14A, E18A, and P24A), except for Ser21 where the sequence coding for Ala and Thr substitution was unstable in *Escherichia coli*; thus, we mutated it to Gly (S21G) and for Gly20, where we intentionally introduced Pro (G20P) to affect the fold of N-terminal region (Fig. 4A).

Viral cell-to-cell spread of mutants D14A, E18A, and P24A was comparable to WT-CP (Fig. 4B and Fig. S6). On the other hand, G20P mutant showed delayed cell-to-cell spread, while S21G mutant remained limited to just single cells at all observed time points (Fig. 4B and Fig. S6).

Next, we performed whole plant imaging to study systemic viral spread of mutants (Fig. 4C and Fig. S8). In accordance with the results of cell-to-cell movement, there were no statistically significant differences in viral multiplication area in systemic tissue when comparing D14A, E18A, or P24A with WT-CP (Fig. 4D, Fig. S5 and S7, and Datasets S8 to S11) at all observed time points. On the other hand, the viral multiplication area of G20P in systemic tissue was statistically significantly lower compared to WT-CP at all observed time points (Fig. 4D and Dataset S8). As expected, S21G did not spread systemically (Fig. 4C). Furthermore, viral assembly was confirmed with electron microscopy in all constructed point mutants, with S21G as an exception, where only oligomeric rings were observed (Fig. S10).

## DISCUSSION

The biological function of C and N-terminal regions of PVY CP has already been initiated in our previous study, where we hypothesized that N-terminal amino acid residues are necessary for an efficient cell-to-cell movement (17). Here, we provide additional insights into the mechanism of PVY spread by identifying the regions of PVY CP N-terminal region that are important for efficient viral cell-to-cell movement and systemic infection.

Despite the conserved flexible structure of potyviruses N-terminal region of CP, there are notable differences observed regarding its involvement in potyviral cell-to-cell and systemic movement. In the case of zucchini yellow mosaic virus (ZYMV), deletion of the entire CP N-terminal region did not affect systemic infection (23). Similarly, a deletion of six to 50 amino acids in the CP N-terminal domain did not compromise TuMV cell-to-cell and systemic movement (19). On the other hand, in tobacco etch potyvirus (TEV), CP N-terminal region deletions of five to 29 amino acid residues delayed cell-to-cell movement and prevented systemic spread (22). We observed an inability to move cell-to-cell for PVY when 40 or more amino acid residues were deleted from the CP N-terminal region (Fig. 1). However, shorter deletions in the range of 19 to 23 did not prevent systemic spread but resulted in delayed systemic and cell-to-cell PVY movement (Fig. 2B and C, Fig. 3B, and Datasets S3, S4, and S7). The observed differences among potyviral species may stem from the high phylogenetic divergence inherent to the genus, which at the CP level is, among other variations, observed as different lengths and nucleotide variations in the N-terminal region across different potyvirus species (23, 29–32). It is also well-known that the potyvirus movement is facilitated by various protein components, both from the virus and the host plant (10, 11, 33–35). In addition, potyviruses exhibit a divergent host range, majority of them being naturally limited to narrow host range, such as TEV and ZYMV (36, 37), while TuMV stands out with its remarkably broad natural host range (38). These features could also be contributing to the differences in the influence that CP N-terminal domain deletions have on the movement of different potyvirus species, because different components involved in potyviral movement, viral and host, could be differently compensating the effect of CP N-terminal deletions.

The analysis of viral multiplication area revealed important insights into the cell-to-cell movement dynamics of deletion mutants, as increasing number of deleted amino acid residues at CP N-terminal region resulted in slower cell-to-cell viral spread (Fig. 2B and C). Furthermore, slower cell-to-cell virus spread also impacted infection of systemic tissue (Fig. 3A). The connection between cell-to-cell movement and systemic spread was expected, as the virus reaches the veins, enters the veins, and is transported in systemic tissue with a delay due to decelerated cell-to-cell movement (39).

Results of several studies showed the role of charged amino acids in the CP core and C- and N-terminal regions of different potyviruses in cell-to-cell and systemic virus spread (20, 26, 40, 41). In contrast, substitutions of charged amino acids in the CP N-terminal region of PVY in our study (D14A and E18A mutants) did not affect cell-to-cell spread (Fig. S6). It was also reported (42) that aromatic residues, located on the core CP region, contribute to the formation of π-stackings that are importantly involved in TVBMV and other potyvirus movement, since they maintain CP accumulation by stabilizing α-helixes and β-sheets (43, 44). Our region of interest does not possess aromatic amino acid residues; therefore, we did not test this hypothesis.

However, we observed that replication of S21G mutant was limited to single cells (Fig. 4B). We further checked, and the intensity of replication of this mutant within this single cell was not affected (Table S2). Interestingly, electron microscopy imaging showed impaired viral assembly, since only oligomeric rings were observed (Fig. S10). Correct viral particle assembly is, however, not a prerequisite for cell-to-cell movement, as ΔN40-CP and ΔN50-CP mutants did assemble but still could not move between cells (Fig. 1 and Fig. S10). We hypothesize that this might be due to loss of potential phosphorylation site or disruption of N-terminus structure, due to introduction of G. Previous studies suggested that post-translational modifications such as phosphorylations have an important role in viral cell-to-cell movement and virion assembly (45, 46). In PVA, phosphorylation of the Thr243 residue on the CP C-terminal region is crucial for PVA replication *in planta* (21). Note that to confirm the hypothesis about the role of phosphorylation of Ser21, we aimed to replace Ser21 also with alanine and threonine, but the design of such point mutants was not achievable, due to their instability in *E. coli*. It has been previously suggested that PVY cDNA is unstable in *E. coli*. Stability of full-length PVY plasmids was achieved by inserting introns into putatively toxic genes, but still, for some mutants recombinations can occur (47).

Furthermore, we also showed the important role of the G on position 20 of CP N-terminal region in PVY cell-to-cell as well as systemic spread, since G20P mutant spreads slower than WT-CP (Fig. 4B through D). We hypothesize that exchanging Gly with Pro in the G20P mutant increased rigidity in the N-terminal region. Consequently, the interaction of the CP with other host and viral proteins in the cell may be impaired, resulting in the observed delay. Moreover, alignment of the first 50 amino acid residues from PVY CP N-terminal region across all PVY isolates revealed a high degree of conservation (at least 92.9%) at the mutated residues 20 (G20P) and 21 (S21G) across all PVY isolates (Fig. S9), further supporting biological significance of these amino acids in PVY.

The results identifying crucial domains and amino acids of CP N-terminal region in viral cell-to-cell movement provided in our study are important basis to elucidate these complex processes, particularly in most economically important viruses such as PVY.

## MATERIALS AND METHODS

### Plant material

*N. clevelandii* plants were used to follow virus spread. Plants were grown from seeds and kept in growth chambers under controlled conditions as described previously (48).

### Construction of PVY-N605(123)-GFP CP N-terminal deletion and point mutants

As a template to construct mutants with deletions on the CP N-terminal region, we used a GFP-tagged infectious clone PVY-N605(123) (28). The GFP coding sequence was inserted between NIb and CP coding sequences, flanked by protease target sequences, so that GFP would not bother viral movement, since it is excised from the polyprotein as other viral proteins (28). Megaprimers were designed in accordance with previously prepared instructions (49). Detailed information about mutagenic PCR reactions conditions and mixtures can be found in Text S1. After adding 4 µL of DpnI enzyme, the mutagenesis mixtures were transformed into *E. coli* XL 10 Ultracompetent Cells in accordance with manufacturer’s instructions (Agilent Technologies). Transformation mixtures were plated on LB agar with ampicillin selection and incubated overnight at 37°C. Transformants were screened with colony PCR using PVY GFP_F and PVY uni_R primers (Dataset S12) and KAPA2G Robust HotStart Kit (Agilent Technologies). Prior to bombardment, polyprotein and its promoter in PVY-N605(123) lacking 50 and 14 amino acids on CP N-terminus were Sanger-sequenced (Dataset S12). Additionally, restriction analysis was performed to confirm the correctness of the PVY coding region.

### Biolistic bombardment

Constructed PVY mutants and wild-type plasmids were amplified in *E. coli*. Plasmids were then isolated and coated onto gold microcarriers that were used for *N. clevelandii* bombardment using a Helios gene gun (Bio-Rad), as described previously (49).

### Plant material sampling and RNA isolation

Plant leaf samples were collected from bombarded *N. clevelandii* plants by excising 0.3 g plant tissue near the bombardment site. Sampled plant material from bombarded *N. clevelandii* leaves was homogenized in 700 µL of RNeasy kit RLT lysis buffer with FastPrep (MP Biomedicals) 1 min 6.5 m/s. Total RNA was isolated using RNeasy Plant Mini Kit (Qiagen). Residual DNA was digested with deoxyribonuclease I (DNase I, Qiagen) in solution, using 1.36U DNase/µg RNA. Quality control of isolated RNA was performed as described (48).

### RT-qPCR

Relative PVY RNA concentration was determined by single-step reverse transcription quantitative PCR (RT-qPCR; AgPath-ID One-Step RT-PCR, Thermo Fisher Scientific) for the ΔN50-CP mutant, while two step RT-qPCR was performed for other mutants. RNA was reverse transcribed with High-Capacity cDNA Reverse Transcription Kit (Thermo Fisher Scientific) and analyzed by qPCR using FastStart Universal Probe Master (Roche).

A qPCR assay targeting the PVY CP encoding region was used as a target gene, while the gene encoding for cytochrome oxidase was used as a reference gene. To avoid false positive results, plants bombarded with plasmid encoding for blue fluorescent protein were sampled and analyzed with our qPCR assay targeting PVY CP encoding region. RT-qPCR amplification program and reaction composition mixtures were the same as previously described (28). Primer and probe details are listed in Dataset S12.

After conducting RT-qPCR reactions, the QuantGenius software (50) was used for relative quantification of PVY RNA using the standard curve approach. Data containing information about the relative RNA quantity were averaged for each group and then normalized to the lowest group average number (Dataset S1).

### RT-PCR

One-Step Reverse Transcription PCR (QIAGEN OneStep RT-PCR Kit) was performed to confirm that the introduced mutations were maintained in the viral progeny of all constructed mutants. DNase-treated RNA, isolated from leaves according to the above protocol, was used as a template. Mutated region was amplified with PVY-GFP_F and uniPVY_R primers (see Dataset S12). The purified PCR products were sequenced (Sanger sequencing). For samples with a small amount of mutated virus (ΔN50-CP, ΔN40-CP, S21G), an additional PCR (repliQa HiFi ToughMix; Quantabio) on purified products was performed to amplify the mutated region in sufficient quantity.

### Confocal microscopy

Cell-to-cell viral spread on upper side of bombarded *N. clevelandii* leaf discs, sampled with cork borer, was observed under Leica TCS LSI confocal macroscope with Plan APO ×5 and ×20 objectives (Leica Microsystems, Wetzlar, Germany) at different time points (5–14 dpb) and Stellaris 5 confocal microscope with HC PL FLUOTAR ×10 objective (Leica Microsystems, Wetzlar, Germany) for studying point mutations at 5 dpb. The GFP emission was recorded after excitation with 488 nm laser in the window between 505 and 530 nm and used as a direct measure of the presence of metabolically active viruses in the observed tissue. Sampled discs were scanned unidirectionally with a scan speed of 400 Hz and a frame average of 1 or 2. For following the spread of ΔN50-CP at 12 dpb, 5× and 20× objectives were used (Fig. 1B), for ΔN40-CP and WT-CP at 5 and 10 dpb, 5× objective was used (Fig. 1B), while for following the spread of ΔN26-CP at 5–14 dpb and ΔN23-CP, ΔN20-CP, ΔN19-CP, and ΔN14-CP mutants and WT-CP at 5 dpb, 20× objective was used (Fig. 1C and D). For each mutant, we observed at least three plants. Images were processed using LEICA LAS X software (Leica Microsystems) to obtain maximum projections from z-stacks. Raw confocal microscopy images were deposited on Zenodo (doi: https://doi.org/10.5281/zenodo.17643798).

### TEM sample preparation

Viral assembly was assessed with transmission electron microscopy (TEM) using negative staining method. Tissue extracts were prepared from leaf discs, in which viral presence was previously confirmed with confocal microscope, and then macerated in phosphate buffer (0.1 M, pH 6.8). Twenty microliters of extracts was applied to a grid for 5 min, blotted, washed, and stained with 1% (wt/vol) water solution of uranyl acetate. Grids were observed by TEM TALOS L120 (Thermo Fisher Scientific) operating at 100 kV or 120 kV. Micrographs were recorded using Ceta 16 M camera and software Velox (Thermo Fisher Scientific). Raw electron microscopy images were deposited on Zenodo (doi: https://doi.org/10.5281/zenodo.17643798).

### Virus inoculation

To more precisely examine cell-to-cell viral movement by whole plant imaging, we inoculated 3- to 4-week-old *N. clevelandii* plants with inoculum prepared from systemic leaves of ΔN14-CP, ΔN19-CP, ΔN23-CP, and WT-CP-inoculated plants in which GFP signal was confirmed using whole plant imaging system. Systemic leaves were ground in phosphate buffer (supplemented with PVP 10000) in a plant material : buffer ratio of 1:4. Three bottom leaves of *N. clevelandii* plants were dusted with carborundum powder and rubbed with the inoculum (one to three drops of inoculum per leaf) which was removed after 10 min by rinsing with tap water.

### Whole plant imaging

Cell-to-cell and systemic viral spread throughout the plant was monitored using the Whole plant imaging system Newton 7.0 BIO (Vilber) in bombarded and inoculated *N. clevelandii* plants. GFP emission of GFP-tagged PVY clone was followed in 480 nm excitation channel and emission filter F-550. Images were taken by EvolutionCapt edge software. For viral multiplication area analysis in inoculated leaves, all three ΔN14-CP-, ΔN19-CP-, ΔN23-CP-, and WT-CP-inoculated leaves of *N. clevelandii* were imaged using exposure time 1 min 46 s 700 ms, 10 × 10 field of view (FOV) and 1,331–1,337 focus in four time points: 3 days post-inoculation (dpi) a.m. and p.m. and four dpi a.m. and p.m. Measurements for a.m. time point were carried out 9 a.m, while for p.m. time point at 1 p.m. Four plants inoculated with each mutant or WT-CP were imaged. For studying systemic viral spread, at least three *N. clevelandii* plants bombarded with ΔN26-CP, ΔN23-CP, ΔN14-CP, ΔN19-CP, and WT-CP were imaged, using 50 s exposure time, 20 × 20 FOV, and focus in the range between 1,871 and 1,905 in different time points (7–12 dpb). Note that *N. clevelandii* plants bombarded with ΔN26-CP and ΔN23-CP were not taken into analysis, due to their inability or big delay in systemic movement. For studying systemic viral spread of point mutations, at least three plants bombarded with constructed point mutants were imaged at different time points (6–13 dpb) with exposure time 50 s and other settings as for studying systemic viral spread of deletion mutants (see above). In the case of the experiment with D14A and WT-CP at 13 dpb, exposure time was only 5 s to avoid saturation due to a high signal. Raw images were deposited on Zenodo (doi: https://doi.org/10.5281/zenodo.17643798).

### Image analysis and signal quantification following whole plant imaging

Viral multiplication area analysis on inoculated leaves and on upper systemic leaves was performed using Kuant software (Vilber, France). In the case of viral multiplication area, surfaces of three viral multiplication areas per leaf were measured, while in the case of viral multiplication area in systemic leaves, total count of the virus-affected area was measured. Total count is determined as a sum of gray values within region of interest (ROI) and is proportional to the intensity and spread of the signal. All measured data from Kuant software were exported to Excel, where total count in ROIs was averaged for each time point and subsequently normalized on the lowest group average number. Normalized viral multiplication areas were statistically evaluated using Welch’s *t*-test and were used for graphic representation.

## Data Availability

Raw confocal microscopy, transmission electron microscopy, whole plant imaging system pictures, and supplemental datasets S1 to S12 were deposited at Zenodo and are openly available at doi: https://doi.org/10.5281/zenodo.17643798.

## AFTER EPUB

*[This article was published on 20 November 2025 with an incorrect figure citation in the Discussion and scale measurements missing from the supplemental material. These items were updated in the current version, posted on 9 December 2025.]*

## SUPPLEMENTAL MATERIAL

Supplemental material jvi.01400-25-s0001.pdf Text S1, Fig. S1 to S10, and Tables S1 and S2.

ASM does not own the copyrights to Supplemental Material that may be linked to, or accessed through, an article. The authors have granted ASM a non-exclusive, world-wide license to publish the Supplemental Material files. Please contact the corresponding author directly for reuse.
