# Supplementary material for: Functionality of potato virus Y coat protein in cell-to-cell movement dynamics is defined by its N-terminal region
Source: J Virol. 2025 Nov 20;99(12):e01400-25. doi: 10.1128/jvi.01400-25 (PMC12724344; doi:10.1128/jvi.01400-25)
Supplement: Supplemental material — Text S1, Fig. S1 to S10, and Tables S1 and S2. [file jvi.01400-25-s0001.pdf]

Supporting Information for

Functionality of potato virus Y coat protein in cell-to-cell movement dynamics is defined by its N terminal region

Anže Vozelj[1,2*], Tjaša Mahkovec Povalej[1], Katja Stare[1], Magda Tušek Žnidarič[1], Katarina Bačnik[1], Valentina Levak[1,2], Ion Gutiérrez-Aguirre[1], Marjetka Podobnik[3], Kristina Gruden[1], Anna Coll[1]†, Tjaša Lukan[1]†[*]

* Corresponding authors. Email: anze.vozelj@nib.si, tjasa.lukan@nib.si

This PDF file includes:

Supplementary Text S1. Construction of PVY-N605(123)- GFP CP N-terminal mutants
Fig. S1. ΔN50-CP ΔN40-CP viral replication.
Fig. S2. Viral replication ΔN26-CP.
Fig. S3. ΔN19-CP_ΔN14-CP_WT-CP cell-to-cell spread dynamics.
Fig. S4. ΔN26-CP inability of systemic spread
Fig. S5. Independent experiment for point mutants G20P, P24A and WT-CP to check viral abundance.
Fig. S6. Cell-to-cell viral spread of D14A, E18A, G20P, S21G and P24A point mutants.
Fig. S7. Virus abundance D14A, E18A, WT-CP.
Fig. S8. Point mutants systemic spread.
Fig. S9. Alignment of first 50 amino acid residues from the PVY N terminal region across all PVY isolates.
Fig. S10. Transmission electron microscopy (TEM) micrographs of deletion and point mutants.
Table S1. ΔN26-CP viral limitation on single cells or cell-to-cell spread.
Table S2. Replication efficiency of ΔN40-CP and S21G mutant is the same as the one of WT-CP PVY.

Other supporting materials for this manuscript are openly available on Zenodo (https://doi.org/10.5281/zenodo.17643798), including the following:

Dataset S1 (Microsoft Excel format). Normalized qPCR data for constructed PVY mutants.
Dataset S2 (Microsoft Excel format). General sample information including sample name, plant and leaf number, date of putting plants into the soil, date of bombardment.
Dataset S3 (Microsoft Excel format). Viral cell-to-cell spread evaluation after *N. clevelandii* inoculation with constructed mutant ΔN23/G-CP and WT-CP.
Dataset S4 (Microsoft Excel format). Viral cell-to-cell spread evaluation after *N. clevelandii* inoculation with constructed mutants ΔN19-CP, ΔN14-CP and WT-CP.
Dataset S5 (Microsoft Excel format). Viral cell-to-cell spread evaluation after *N. clevelandii* inoculation with constructed mutants ΔN19-CP, ΔN14-CP and WT-CP in replicate experiment.
Dataset S6 (Microsoft Excel format). Foci analysis comparisons between experiments.
Dataset S7 (Microsoft Excel format). Systemic spread dynamic analysis.
Dataset S8 (Microsoft Excel format). Systemic viral spread of G20P and P24A point mutants.
Dataset S9 (Microsoft Excel format). Systemic viral spread of G20P and P24A point mutants in replicate experiment.
Dataset S10 (Microsoft Excel format). Systemic viral spread of D14A point mutant.
Dataset S11 (Microsoft Excel format). Systemic viral spread of E18A point mutant.
Dataset S12 (Microsoft Excel format). Primers and megaprimers sequences.

Supporting Information Text S1. Construction of PVY-N605(123)- GFP CP N-terminal mutants.

Mutants were prepared with mutagenic PCR using QuikChange II XL Site-Directed Mutagenesis Kit (Agilent Technologies). As a template previously constructed GFP infectious clone PVY-N605(123) was used (1). The megaprimers were synthesized using the N-terminal region sequence of GFP tagged PVY-N605(123) plasmid, according to defined guidelines (2). All megaprimers used in the study are listed in dataset S12. Mutagenic touchdown PCR reaction program with the following reaction mixture in the final volume of 25 μL were the same for all generated mutants according to previously published protocol for generation of PVY deletion mutants (2), with minor modifications listed below.

**Component**	**Final concentration**	**Volume [μL]**
10x reaction buffer	1x	2,5
dNTP mix	200 μM	0,5
forward megaprimer	0,5 μM	1,25
reverse megaprimer	0,5 μM	1,25
QuikSolution	/	1,5
PVY-N605(123)	200 ng	1,2
PfuUltra HF DNA polymerase	/	1

**Temperature**	**Time**	**Step**
92ºC	2 min	hold
92ºC	50 s	
65ºC to 55ºC	50 s	10cycles
68ºC	30 min*	
92ºC	50 s	
55ºC	50 s	8 cycles
68ºC	30 min*	
68ºC	60 min	hold

After amplification, 4 µL of DpnI enzyme (Agilent Technologies) was added to the mutagenesis reaction mixture, following 2h on 37ºC incubation. Following DpnI digestion, 2 µL of mutagenesis mixture was used for transformation into *E. coli* XL-10 Gold Ultracompetent Cells (Agilent Technologies). We used 45 µL cell aliquot supplemented with 2 µL of β-mercaptoethanol for the standard heat-shock transformation protocol in accordance with the manufacturer's instructions (Agilent Technologies). Transformation mixtures were plated on LB agar containing ampicillin and incubated overnight at 37ºC. Transformants were analyzed with colony PCR using primers PVY GFP_F and PVY uni_R with KAPA2G Robust HotStart Kit (Agilent Technologies) with the following 10 µL reaction mixture and cycling conditions stated below.

Component	Final concentration	Volume [µL]
5x Buffer B	1x	2
10 mM dNTP	200 µM	0,2
10 µM PVY GFP_F	300 nM	0,3
10 µM PVY uni_R	300 nM	0,3
5 U/µL KAPA2G polymerase	0,3 U	0,06
Colony suspension	/	1
H20		6,14

Temperature	Time	Step
95 °C	10min	Hold
95 °C	30 s	
55 °C	15 s	30 cycles
72 °C	1 min	
72 °C	5 min	Hold

Sanger sequencing, using the same primers as for the colony PCR, of positive colonies was performed to confirm correct sequence of the PVY coding part.

Designed mutants PVY-N605(123)-GFP with desired mutations were amplified in One Shot® TOP10 *E. coli* and 50 µg of constructed plasmid mutants were isolated from overnight cultures using GenElute Plasmid MiniPrep Kit (Sigma-Aldrich). Isolated plasmids were subsequently used to coat 6.25 mg of gold microcarriers (0,6 µm) to prepare gene gun bullets according to the manufacturers protocol and were used for *Nicotiana clevelandii* bombardment using a Helios®gene gun (Bio-Rad) at 200 psi (2).

Supporting figures and tables

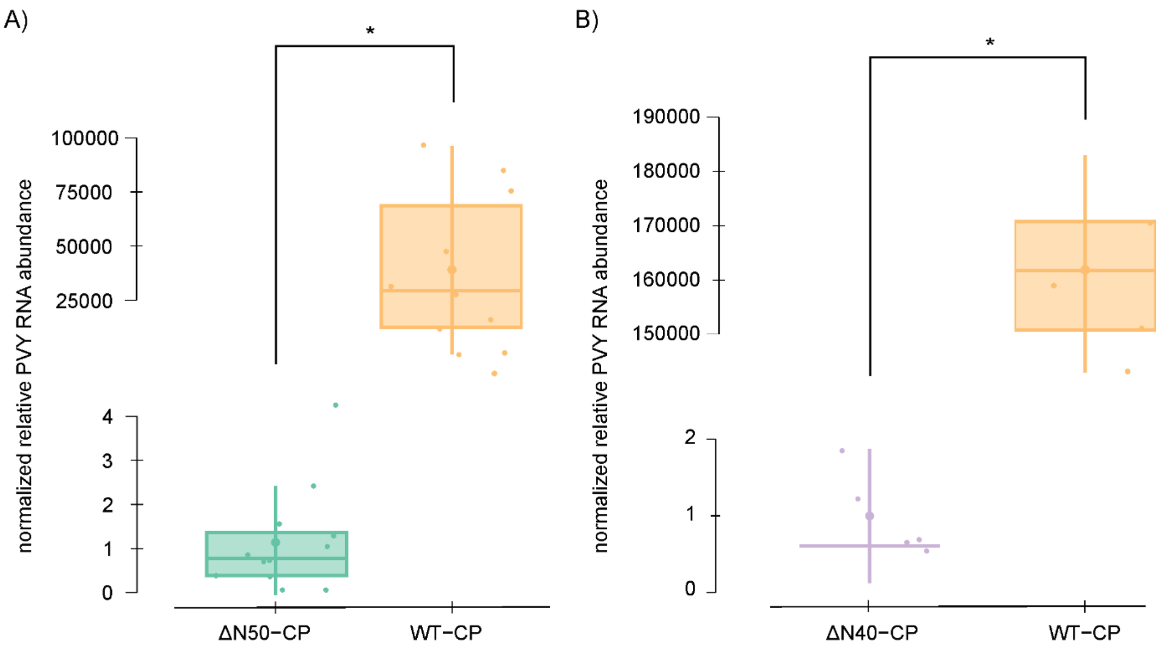

Fig. S1. ΔN50-CP ΔN40-CP viral replication. Normalized relative PVY RNA abundance in bombarded *N. clevelandii* leaves for constructed PVY mutants lacking 50 (ΔN50-CP) (A) and 40 (ΔN40-CP) (B) amino acids at CP N-terminus. Results were obtained 14 days post bombardment (dpb). Non-mutated infectious clone (WT-CP) was used as a control. Data normalization was performed as described in dataset S1. Results are presented as boxplots with normalized relative PVY RNA abundance for each sample shown as dots. Differences between ΔN50-CP and WT-CP and between ΔN50-CP and WT-CP were statistically evaluated using Welch's t test. Statistically significant differences (p<0,05) are marked with an asterisk (*). Vertical lines present all points except outliers.

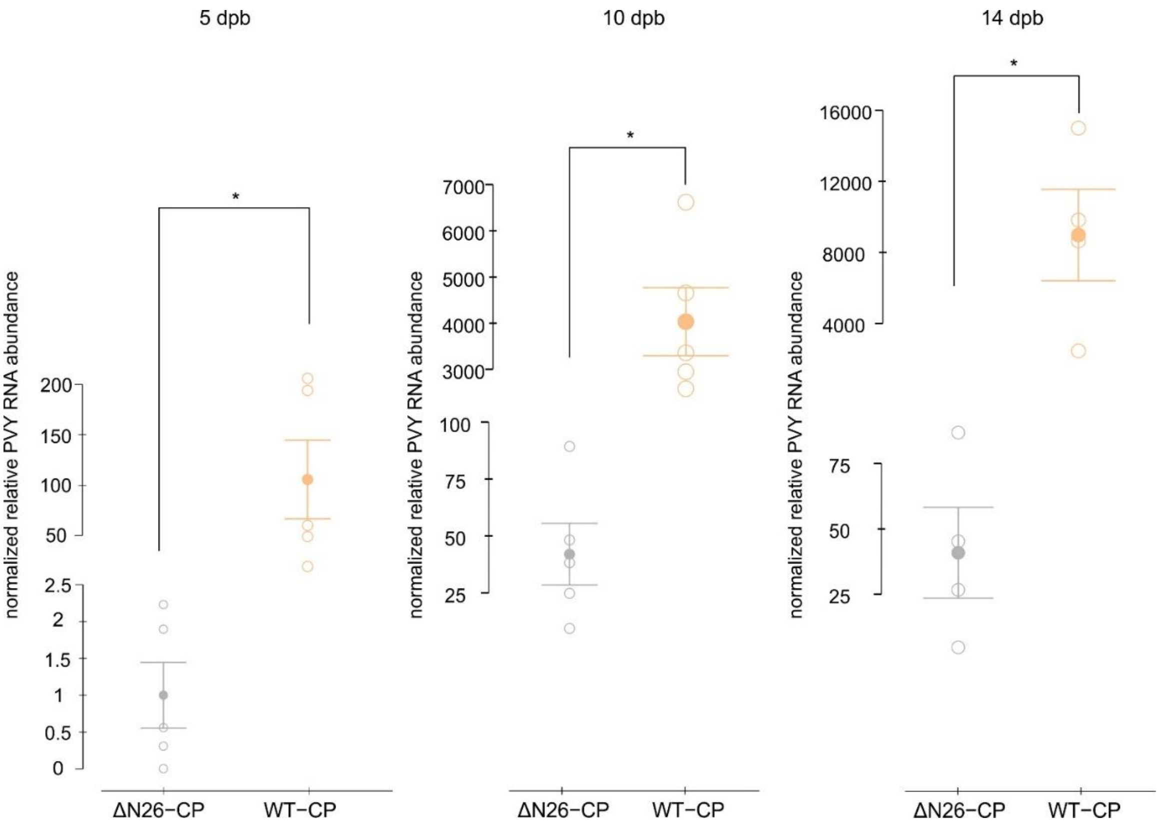

Fig. S2. Viral replication ΔN26-CP. Normalized relative PVY RNA abundance in bombarded *N. clevelandii* leaves for constructed PVY mutant lacking 26 amino acids residues, in three timepoints including 5, 10 and 14 dpb (from left to right). Non-mutated infectious clone (WT-CP) was used as a control. Data normalization was performed as described in dataset S1. Results are presented as mean (represented with filled dot) and standard error. Individual measurements are shown as empty dots, representing normalized relative PVY RNA abundance. Differences between constructed deletion mutants and WT-CP were statistically evaluated using Welch's t test. Statistically significant differences (p<0,05) are marked with an asterisk (*). Note that the scales are different between time points.

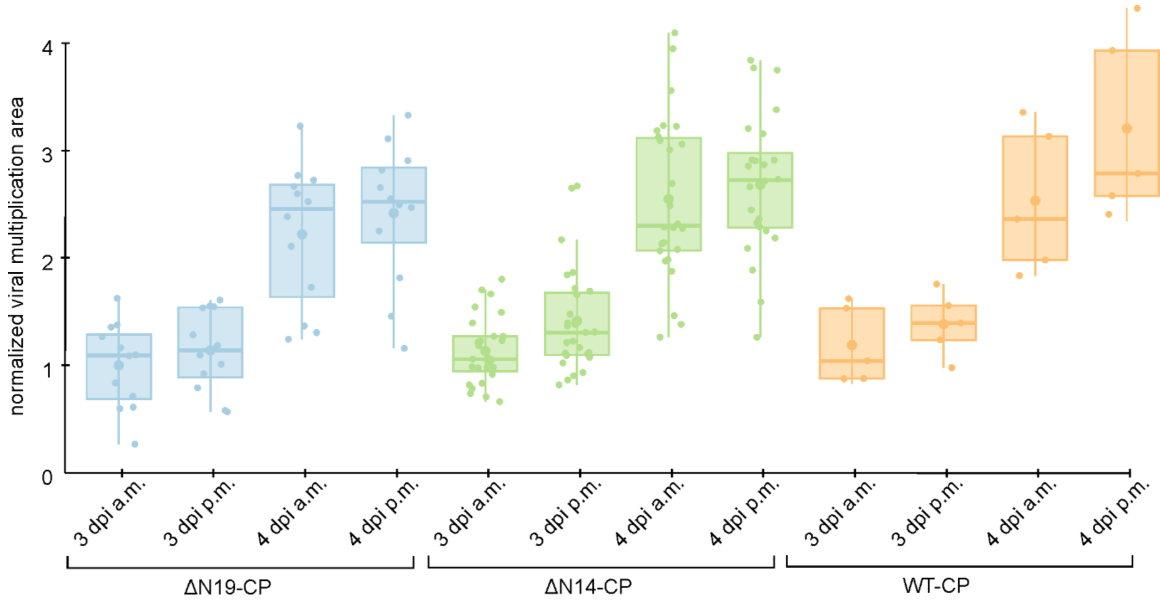

Fig. S3. N19-CP_ΔN14-CP_WT-CP cell-to-cell spread dynamics. Viral cell-to-cell spread dynamics was quantified with normalized viral multiplication analysis as described in Materials and methods. Results are presented as boxplots for tested mutants ΔN19-CP, ΔN14-CP and WT-CP in 4 tested timepoints including 3 dpi a.m., 3 dpi p.m., 4 dpi a.m. and 4 dpi p.m., where dots are representing normalized viral multiplication area as described in dataset S5. Differences were statistically evaluated using Welch`s t test. Vertical lines present all points except outliers. The differences were not statistically significant, due to autofluorescence of trichomes which resulted in saturated pixels.

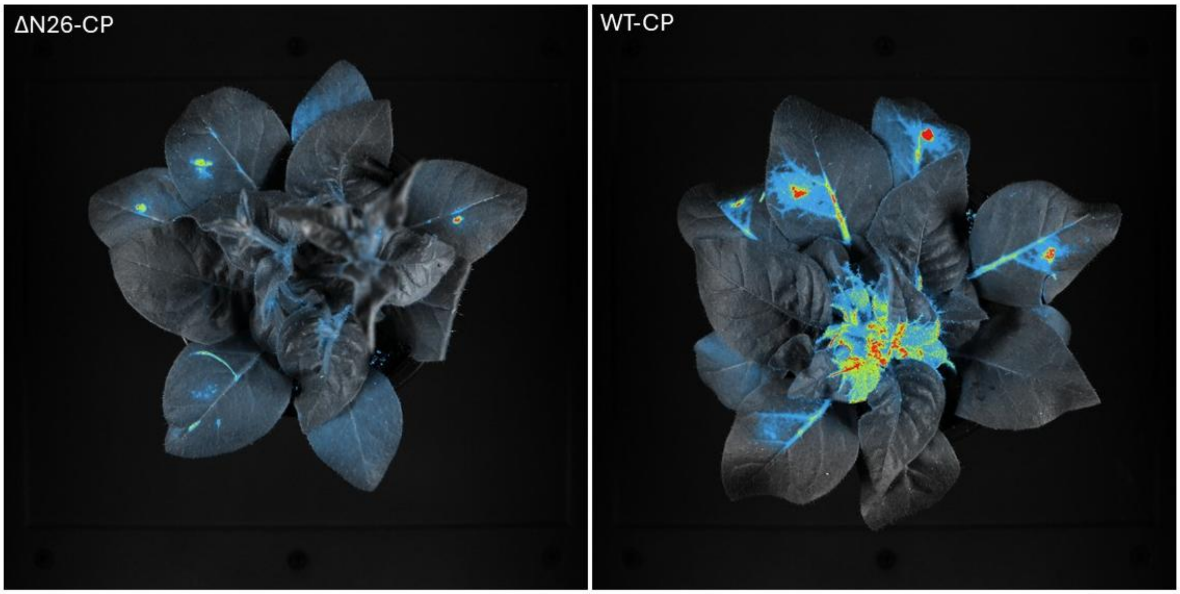

Fig. S4. ΔN26-CP inability of systemic spread. Viral systemic spread was abolished in ΔN26-CP mutant (left) in comparison to non mutated WT-CP, where systemic spread occurred (right). Pictures taken 12 dpb.

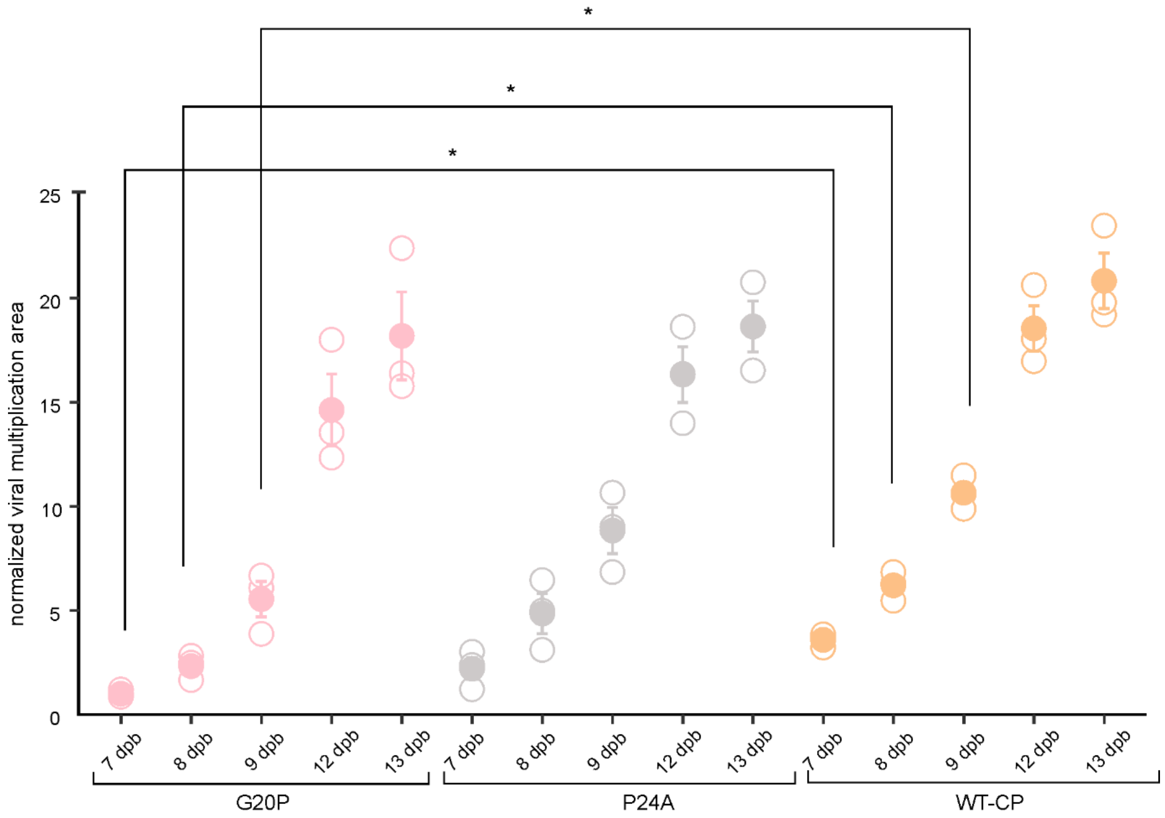

Fig. S5. Independent experiment for point mutants G20P, P24A and WT-CP to check viral abundance. Quantification of virus abundance in the upper leaves of bombarded *N. clevelandii* expressed as total count per mutant 7, 8, 9, 12 and 13 dpb with exposure time 50 s (A). Other measurements settings are the same as stated in Materials and methods. Mean (represented with filled dot) and standard error are shown. Individual measurements are shown as empty dots, representing normalized viral multiplication area. Statistically significant difference in normalized viral multiplication area between mutants was evaluated by Welch`s t test. Statistically significant differences (p<0,05) are demarked with an asterisk (*). Raw and normalized data, number of plants and results of statistical analysis are specified in dataset S9. Note that there was no statistically significant difference between G20P and WT-CP at 12 and 13 dpb due to signal oversaturation due to high exposure time (50 s).

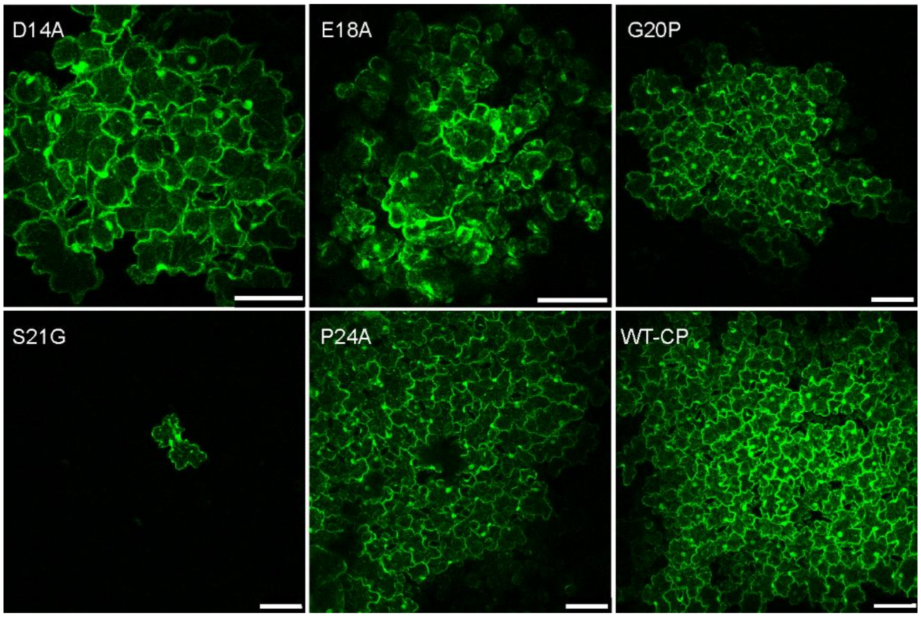

Fig. S6. Cell-to-cell viral spread of D14A, E18A, G20P, S21G and P24A point mutants. Confocal microscopy images showing viral cell-to-cell spread of D14A, E18A, G20P, S21G, P24A point mutants and WT-CP 5 dpb. Note that there we have comparisons of D14A and E18A point mutants with the others (G20P, S21G, P24A) already included in the article main text (Fig. 4B). Other confocal microscopy settings are specified in Materials and methods. Scale is 100 µm.

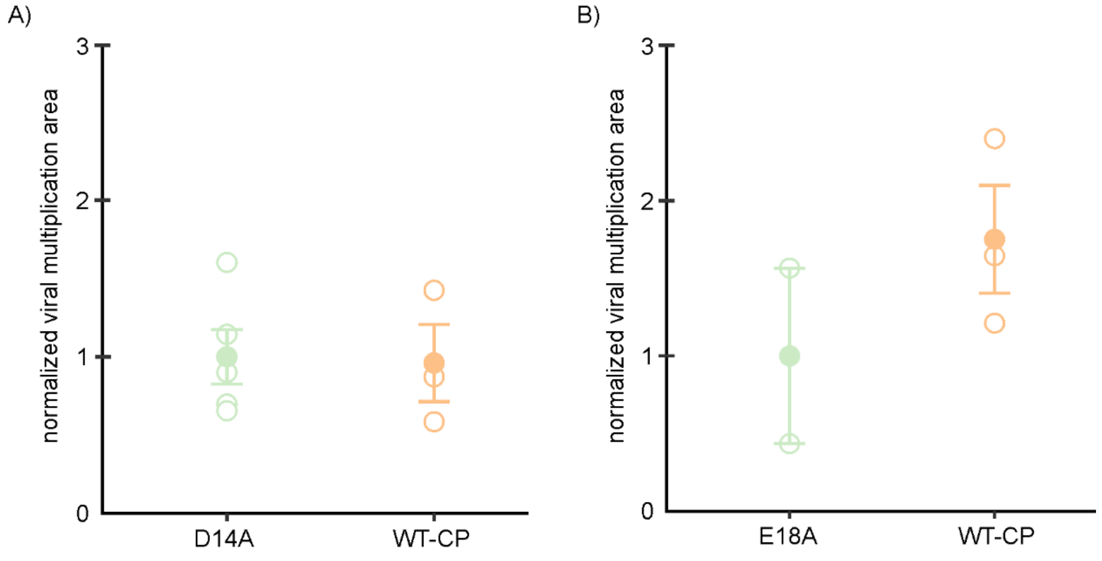

Fig. S7. Virus abundance D14A, E18A, WT-CP. Quantification of virus abundance in the upper leaves of bombarded *N. clevelandii* expressed as total count per mutant for D14A mutant 7 dpb with exposure time 50s (A) and (B) for E18A mutant 7 dpb with exposure time 50 (s). Other measurements settings are the same as stated in Materials and methods. Mean (represented with filled dot) and standard error are shown. Individual measurements are shown as empty dots, representing normalized viral multiplication area. There was no statistically significant difference in normalized viral multiplication area between mutants evaluated by Welch`s t test. Raw and normalized data, number of plants and results of statistical analysis are specified in dataset S10 and S11).

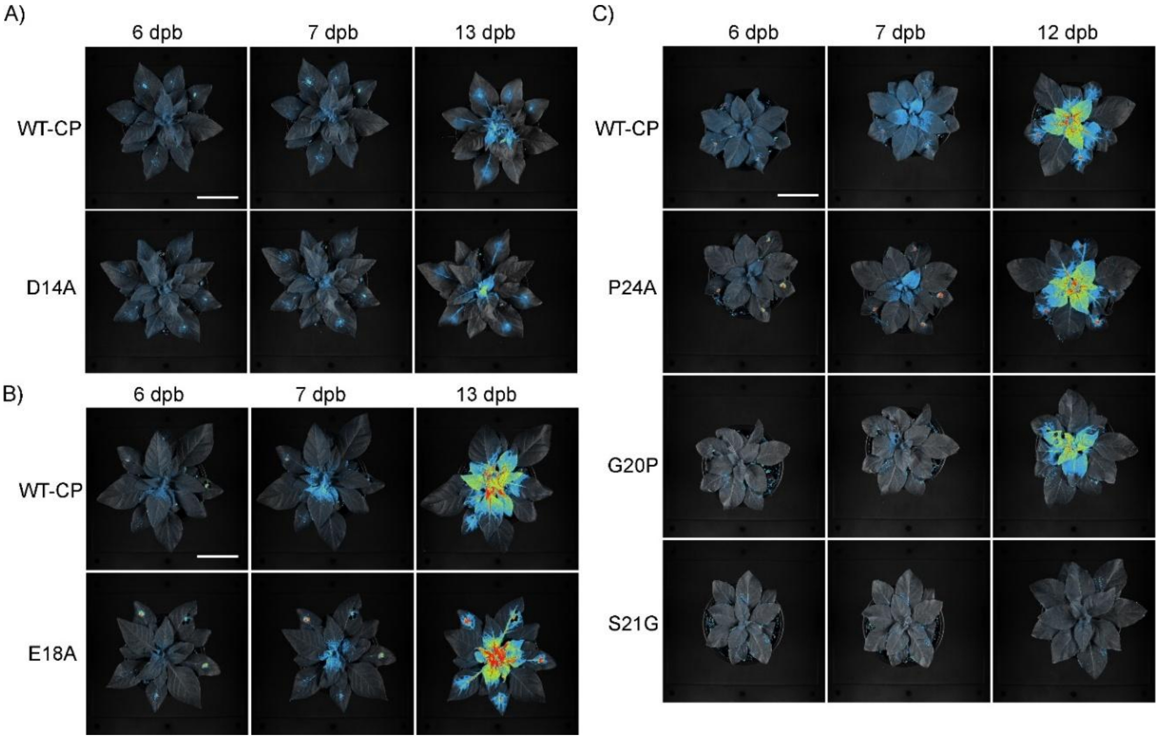

Fig. S8. Point mutants systemic spread. Spatio-temporal PVY distribution in *N. clevelandii* systemic tissue using whole plant imaging system. Systemic spread of constructed point mutants was followed 6 dpb, 7 dpb and 13 dpb in case of D14A (A) and E18A (B), while systemic spread of P24A, G20P and S21G. (C) was followed 6 dpb, 7 dpb and 12 dpb. Note that pictures for P24A, S21G and WT-CP 12 dpb are the same as in the main text (Fig. 4C). Imaging settings are specified in Materials and methods. Plants were imaged with exposure time 50 s. In case of D14A and WT-CP at 13 dpb, exposure time was 5 s to avoid oversaturation due to a higher signal. Scale is 5 cm.

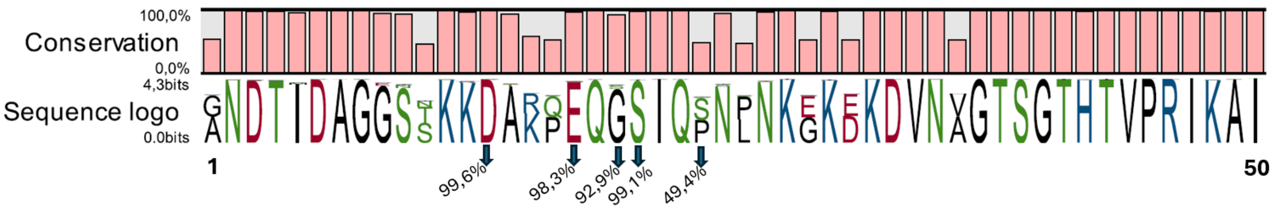

Fig. S9 Alignment of first 50 amino acid residues from the PVY N terminal region across all PVY isolates. To assess the conservation of mutated amino acid residues D14A, E18A, G20P, S21G and P24A, we performed multiple sequence alignment of the first 50 amino acid residues of the PVY coat protein, using CLC Genomics Workbench 25 (QIAGEN, Hilden, Germany) and pairwise sequence alignment. Note that for the multiple alignment analysis, only complete sequences containing the first 50 amino acid residues of the PVY coat protein N terminal region were included, as this region corresponds to our engineered deletion and point mutants. Altogether 2112 sequences were obtained from NCBI Virus database. Sequence logo represents the amino acid sequence conservation in the mutated region with arrows showing the conservation percentage of each point mutated amino acid (D14A, E18A, G20P, S21G, P24A) across all obtained sequences.

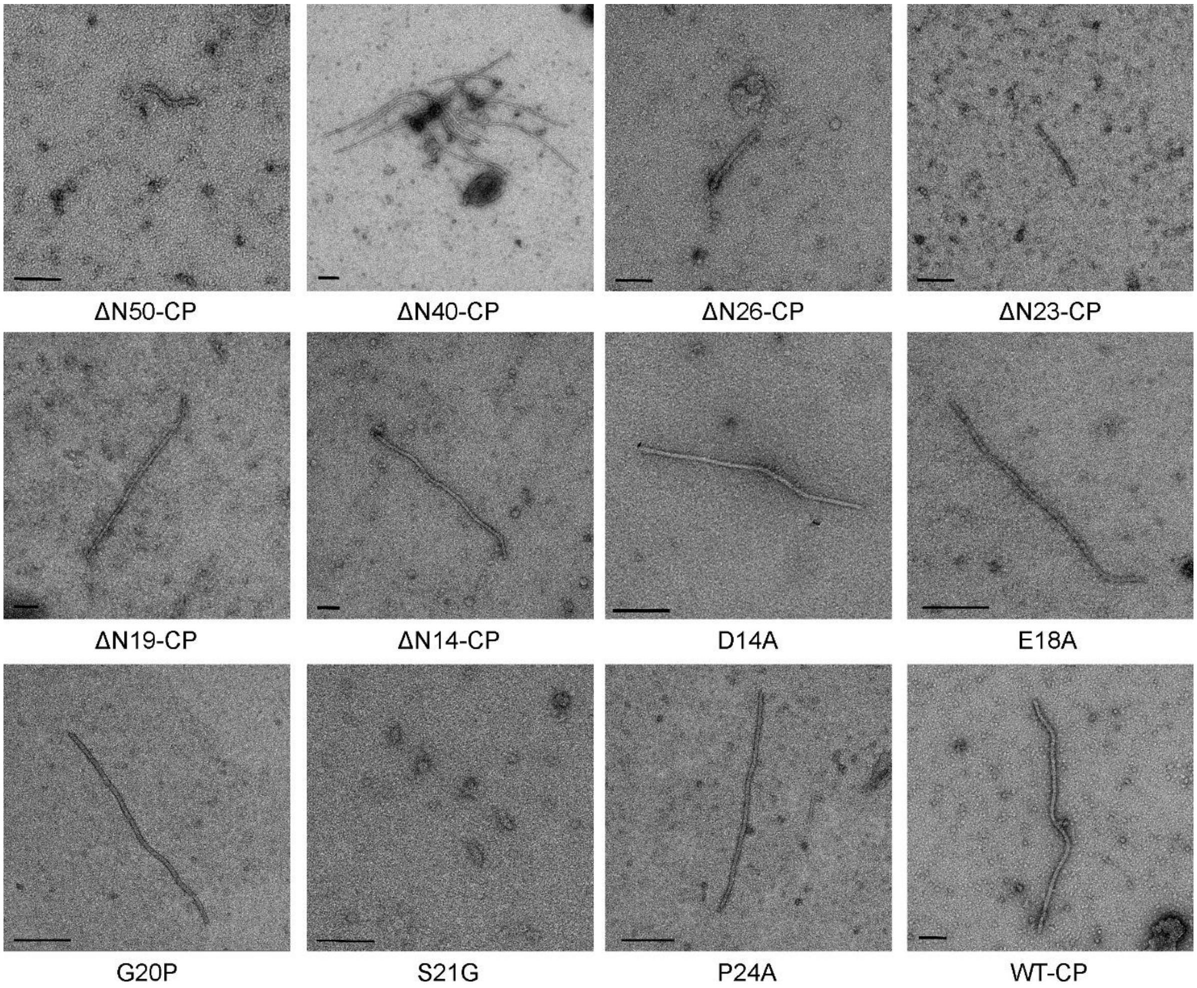

Fig. S10 Transmission electron microscopy (TEM) micrographs of deletion and point mutants. Representative TEM micrographs of deletion mutants and point mutants. Results were obtained with negative staining. Scale bars for deletion mutants and WT-CP are 100 nm and for point mutations 200 nm except in case of S21G (50 nm). Additional images of the mutant viruses were deposited at Zenodo (doi: 10.5281/zenodo.17643798).

Table S1. ΔN26-CP viral limitation on single cells or cell-to-cell spread. Number of plants with viral cell-to-cell spread or viral limitation to single cells 10 and 14 dpb. Note that 5 dpb virus was limited to single cells in all observed plants.

		**ΔN26-CP**	**WT-CP**
**10 dpb**	single cells	3/8	0/2
	cell-to-cell	5/8	2/2

		**ΔN26-CP**	**WT-CP**
**14 dpb**	single cells	2/3	0/2
	cell-to-cell	1/3	2/2

Table S2. Replication efficiency of ΔN40-CP and S21G mutant is the same as the one of WT-CP PVY. To confirm that detected fluorescent signal in ΔN40-CP and S21G PVY mutants, was the consequence of viral replication and not the continuous expression of viral genes from the original plasmid of PVY driven by the constitutive 35S promoter, ROI (regions of interests) mean intensities of individual cells in confocal microscopy images were assessed. Mean intensities in selected ROIs 5 dpb for ΔN40-CP (A) and S21G (B) compared to WT-CP PVY are shown. Statistical significance of differences was evaluated using Welch`s t test. Note that all images were taken using the same settings (objective, zoom, gain).

A)

**ΔN40-CP**	**WT-CP**	**Welch`s t test**
24.4	13.8	0.6
14.0	17.1	
9.7	15.5	
13.3	9.4	
8.4	15.2	
4.8	21.6	
11.5	13.7	
8.6	15.6	
13.1	8.7	
25.7	15.9	
**13.3**	**14.6**	**average**

B)

**S21G**	**WT-CP**	**Welch`s t test**
6.8	7.1	0.2
6.8	9.4	
6.8	12.8	
**6.8**	**9.8**	**average**

Supplemental material references

1. Lukan T, Županič A, Mahkovec Povalej T, Brunkard JO, Kmetič M, Juteršek M, Baebler Š, Gruden K. Chloroplast redox state changes mark cell-to-cell signaling in the hypersensitive response. New Phytologist. 2023; 237(2): 548-562.

2. Stare K, Coll A, Gutiérrez-Aguirre I, Žnidarič MT, Ravnikar M, Kežar A, Kavčič L, Podobnik M, Gruden K. Generation and in Planta Functional Analysis of Potato Virus Y mutants. Bio Protoc. 2020; 10: e3692.

3. Kežar A, Kavčič L, Polák M, Nováček J, Gutiérrez-Aguirre I, Žnidarič MT, Coll A, Stare K, Gruden K, Ravnikar M, Pahovnik D, Žagar E, Merzel F, Anderluh G, Podobnik M. 2019. Structural basis for the multitasking nature of the potato virus Y coat protein. Sci Adv 5:eaaw3808. https://doi.org/10.1126/sciadv.aaw3808.

4. Kogovšek P, Gow L, Pompe-Novak M, Gruden K, Foster GD, Boonham N, Ravnikar M. 2008. Single-step RT real-time PCR for sensitive detection and discrimination of Potato virus Y isolates. J Virol Methods 149:1–11. https://doi.org/10.1016/j.jviromet.2008.01.025.

5. Weller SA, Elphinstone JG, Smith NC, Boonham N, Stead DE. 2000. Detection of Ralstonia solanacearum strains with a quantitative, multiplex, real-time, fluorogenic PCR (TaqMan) assay. Appl Environ Microbiol 66:2853–2858. https://doi.org/10.1128/AEM.66.7.2853-2858.2000
